# A Logistic Regression Model Integrating Flow Cytometry−Derived Immune Cell Profiles and Hematological Parameters for Preoperative Prediction of Peritoneal Metastasis in Gastric Cancer

**DOI:** 10.3390/cancers18172813

**Published:** 2026-08-30

**Authors:** Ruihu Zhao, Yuming Ju, Zhichao Yu, Yingwei Xue, Hongjiang Song

**Affiliations:** Department of Gastrointestinal Surgery, Harbin Medical University Cancer Hospital, Harbin Medical University, Harbin 150081, Chinaxueyingwei@hrbmu.edu.cn (Y.X.)

**Keywords:** gastric cancer, peritoneal metastasis, logistic regression, NK cells, NKT−like cells

## Abstract

Background: Peritoneal metastasis (PM) is a lethal and often occult event in advanced gastric cancer (GC), and its preoperative detection remains difficult. The role of peripheral NK and NKT−like cells in predicting PM has not been well defined. Methods: We retrospectively analyzed 433 patients who underwent surgery for GC from 2016 to 2020. Patients were stratified by PM status and randomly assigned to training and held−out internal validation cohorts. Flow cytometry−derived lymphocyte subset percentages were treated as compositional data, and NK and NKT−like cell variables were entered into the model after log−ratio transformation. Candidate variables were selected using LASSO logistic regression in the training cohort and further assessed by multivariable logistic regression. Model performance was evaluated using AUROC, AUPRC, and confusion matrices in the validation cohort. Nomogram and SHAP analyses were used for model interpretation. Results: Seven predictors were retained in the final model: log−ratio−transformed NKT−like cells, direct bilirubin, prealbumin, lymphocyte count, lactate dehydrogenase, CA125, and log−ratio−transformed NK cells. The model achieved an AUROC of 0.879 (95% CI: 0.805–0.936) and an AUPRC of 0.698 (95% CI: 0.524–0.848) in the validation cohort. At the conventional threshold of 0.50, the model achieved an accuracy of 0.915, sensitivity of 0.821, specificity of 0.941, PPV of 0.793, and NPV of 0.950. At the Youden−optimal threshold of 0.101, sensitivity, specificity, accuracy, PPV, and NPV were 1.000, 0.892, 0.915, 0.718, and 1.000, respectively. Conclusions: A preoperative model integrating log−ratio−transformed NK/NKT−like cell variables and routine hematological parameters showed good ability to identify PM in GC and may help select patients for diagnostic laparoscopy or closer preoperative evaluation.

## Simple Summary

Peritoneal metastasis is a clinically challenging manifestation of advanced gastric cancer and is often difficult to detect before surgery. In this study, preoperative blood parameters from 433 patients were analyzed to predict synchronous peritoneal metastasis already present at initial staging or surgery, rather than postoperative peritoneal recurrence. Because flow cytometry−derived lymphocyte subset percentages are compositional data, NK and NKT−like cell variables were incorporated into the prediction model after log−ratio transformation. The final logistic regression model integrated log−ratio immune−cell variables with routine hematological parameters and showed good discriminative performance in a held−out internal validation cohort. A nomogram and SHAP analysis were used to support individualized risk estimation and model interpretation. The model may assist clinicians in identifying patients who could benefit from diagnostic laparoscopy or intensified evaluation, but it should not replace pathological confirmation, diagnostic laparoscopy, or direct surgical exploration.

## 1. Introduction

Gastric cancer (GC) is among the malignancies with the highest global incidence and mortality rates and is one of the most common gastrointestinal cancers worldwide [1]. According to GLOBOCAN 2022, GC ranks fifth in global cancer incidence, accounting for 4.9% of all new cancer cases; notably, its incidence among both men and women in China is significantly higher than the global average [2]. Localized GC can be treated with surgery, immunotherapy, and chemotherapy. However, the 5−year survival rate among patients with metastatic GC remains extremely low, at less than 10% [3]. Peritoneal metastasis (PM) is the most frequent pattern of metastasis and recurrence in advanced GC. Approximately 60% of patients with GC die of PM [4]; among patients with advanced GC and PM, the median survival is only 3–6 months, and the 5−year survival rate is less than 2% [5]. Currently, diagnostic laparoscopy combined with peritoneal lavage cytology remains the gold standard for detecting PM in patients with GC. However, because of the extensive surface area and complex folds of the parietal and visceral peritoneum, laparoscopy often fails to provide a comprehensive assessment.

Serum tumor markers, including carcinoembryonic antigen and CA19−9, as well as systemic inflammatory markers such as C−reactive protein and host immune markers, have been investigated as potential indicators of postoperative PM in GC [6]. However, their diagnostic sensitivity and positive predictive value remain limited, suggesting that hematological markers alone are insufficient for diagnosing PM. Recent studies have reported that the NLR−PLR−DDI score, a composite index based on multiple hematological parameters, can predict the peritoneal cancer index and prognosis in patients with GC and PM [7]. Nevertheless, most previous studies have focused on isolated markers, and few have developed comprehensive indices that incorporate the features of the immune microenvironment.

In addition to these traditional biomarkers, NK cells have attracted increasing interest because of their prognostic and predictive relevance in cancer. NK cells are innate lymphoid cells that can induce target cell apoptosis without prior antigen presentation, primarily through perforin− and granzyme B−mediated cytotoxicity. Evidence from both mouse models and patients with GC suggests that NK cells participate in immune surveillance against primary tumor formation, local recurrence, and distant metastasis [8,9]. However, in advanced tumors, tumor−infiltrating NK cells frequently become functionally exhausted [10]. This impairment is shaped by multiple endogenous and exogenous factors within the tumor microenvironment. Among these factors, cellular metabolism appears to play an important role in regulating immune cell fate and function. Nutrient scarcity in the tumor microenvironment limits NK cell metabolic activity, thereby compromising NK cell effector function [11,12].

At present, the prediction of peritoneal metastasis is largely dependent on imaging findings and tumor markers, which may fail to detect occult PM in a subset of patients. To the best of our knowledge, this is the first study to develop an integrated composite index that combines hematological parameters with lymphocyte subsets to identify patients at high risk of peritoneal metastasis in GC. We further constructed a PM risk prediction model using LASSO logistic regression to reduce the effect of class imbalance during model training. This model is designed to complement existing staging modalities by identifying patients with negative or equivocal imaging findings who may harbor occult PM and would benefit from diagnostic laparoscopy or intensified staging workup. By closely integrating model visualization with clinical practice, this study provides a supplementary clinical decision support tool for the early, non−invasive risk stratification of peritoneal metastasis in GC, rather than serving as a definitive diagnostic instrument or replacement for the gold standard of surgical or pathological confirmation.

## 2. Materials and Methods

### 2.1. Study Population and Data Collection

This retrospective study included 433 patients who underwent surgery for GC at the Harbin Medical University Cancer Hospital between January 2016 and December 2020. Clinical data were extracted from electronic medical records. Access to patient information followed relevant data protection and privacy regulations, and all personal data were handled in accordance with the ethical principles of the Declaration of Helsinki (Ethics Number: 2020−103). Patients were included if they met the following criteria: (1) pathological diagnosis of GC; (2) surgical respectability confirmed by at least three attending physicians at the level of Associate Chief Physician or above; and (3) sufficient physical condition to tolerate surgery.

**Figure 1:**
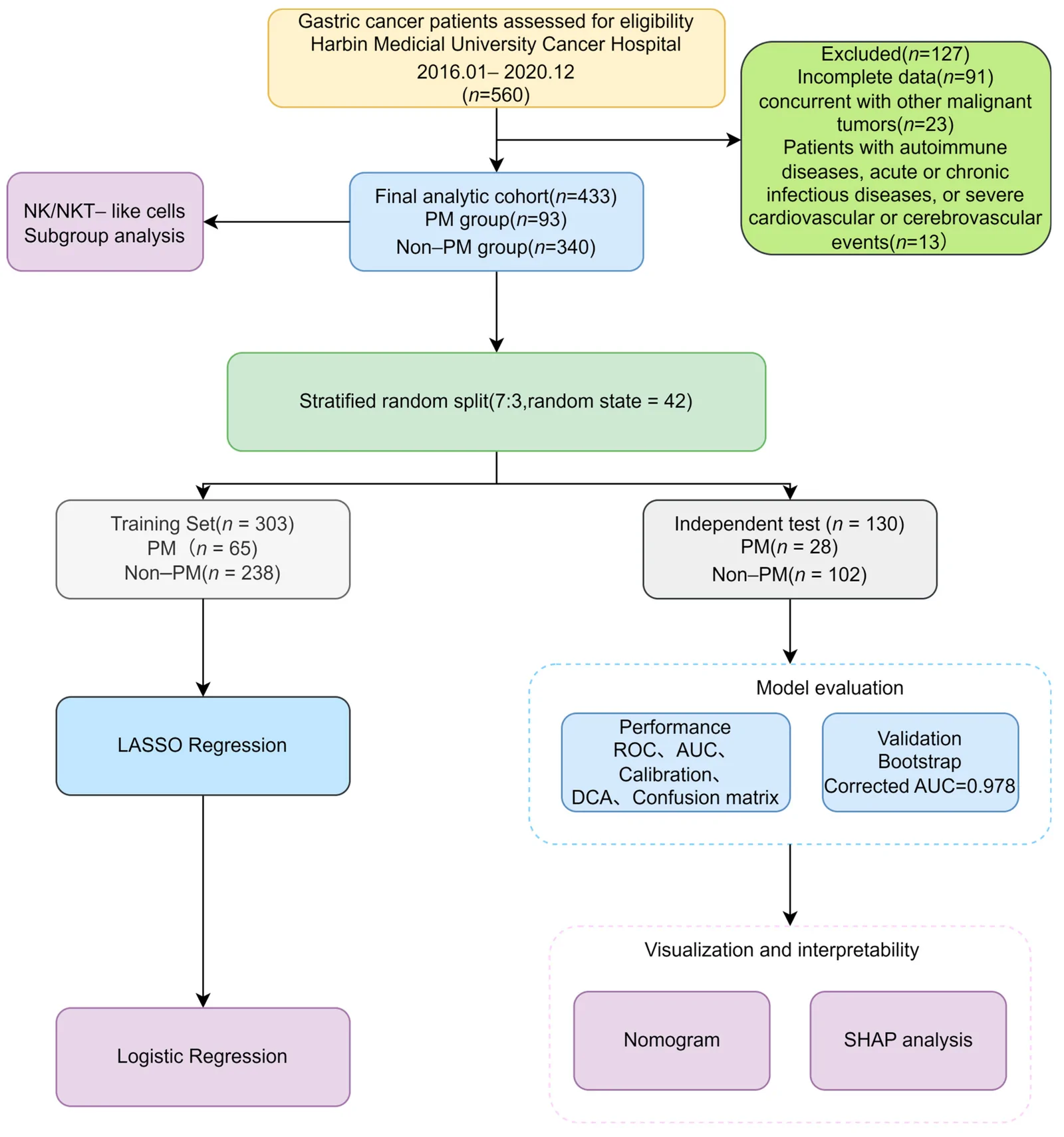
Study design and patient selection flowchart.

Patients were excluded if they had the following conditions: (1) incomplete clinical data, especially missing preoperative blood test results; (2) concurrent malignancies; or (3) autoimmune disease, acute or chronic infection, or severe cardiovascular or cerebrovascular events (Figure 1).

### 2.2. Definition and Determination of Peritoneal Metastasis

The primary outcome of this study was synchronous peritoneal metastasis (PM) identified during the initial surgical exploration. PM was defined as macroscopically visible metastatic lesions involving the parietal or visceral peritoneum detected during intraoperative abdominal exploration. Patients were classified into the PM group when peritoneal metastatic nodules were identified by the operating surgeons during exploration. When clinically and technically feasible, suspicious peritoneal lesions were biopsied for histopathological confirmation.

PM status in the present study was determined exclusively on the basis of macroscopic findings during surgical exploration. Accordingly, the outcome predicted by the model was synchronous macroscopic PM present at the time of the initial operation, rather than postoperative peritoneal recurrence or cytology−only peritoneal dissemination.

All included patients underwent abdominal exploration before a definitive surgical decision was made. During exploration, the abdominal and pelvic cavities were systematically inspected for evidence of peritoneal dissemination, including the diaphragmatic surfaces, liver surface, greater and lesser Omentum, paracolic gutters, mesentery, pelvic peritoneum, and Douglas pouch. Patients without evidence of PM subsequently underwent gastrectomy according to the planned surgical strategy. In patients in whom PM was identified intraoperatively, curative gastrectomy was not performed, and the procedure was limited to exploratory laparotomy.

Peripheral venous blood samples used for hematological and flow cytometric analyses were obtained at the first blood collection after hospital admission and before surgery. The interval between blood sampling and the intraoperative determination of PM status was calculated from the date of the initial blood collection to the date of surgery.

### 2.3. Flow Cytometric Analysis of Lymphocyte Subsets

All patients underwent peripheral blood lymphocyte subset analysis before surgery. Peripheral venous blood samples were obtained during the first routine blood collection after hospital admission and before any surgical intervention. Briefly, 100 μL of fasting peripheral blood was collected and processed according to the manufacturer’s protocol. TBNK reagent (Becton, Dickinson and Company, Franklin Lakes, NJ, USA) was added, followed by vortex mixing and incubation for 30 min at room temperature in the dark. Red blood cell lysing solution was then added, and the samples were incubated for an additional 15 min. After centrifugation, the supernatant was discarded, and the cell pellet was resuspended in phosphate−buffered saline.

Flow cytometric acquisition was performed using a BD FACSCanto II flow cytometer (Becton, Dickinson and Company, BD Biosciences, San Jose, CA, USA), and data were analyzed using BD FACSCanto clinical software (Becton, Dickinson and Company, BD Biosciences, San Jose, CA, USA, version 2.4). Lymphocyte subsets were reported as percentages of gated lymphocytes according to standardized procedures in the institutional clinical laboratory.

Flow cytometry−derived lymphocyte subset variables were expressed as percentages of gated lymphocytes and therefore represented compositional data. Because such variables are constrained by the total lymphocyte composition, raw percentages of multiple lymphocyte subsets were not directly and simultaneously entered into the regression model without preprocessing. To reduce the potential bias caused by the constant−sum constraint, lymphocyte subset variables were first inspected for completeness and plausibility. The immune−cell composition was then constructed using mutually interpretable lymphocyte components, including NK cells, NKT−like cells, CD19+ B cells, and the remaining lymphocyte fraction. The remaining lymphocyte fraction was calculated as 100% minus the sum of the included lymphocyte subsets. When zero values were present, a small−value replacement strategy was applied before log−ratio transformation.

After closure of the lymphocyte composition, log−ratio transformation was performed to allow the lymphocyte subset variables to be used appropriately in regression analysis. Specifically, transformed immune−cell variables were generated to represent the relative abundance of NK cells and NKT−like cells within the lymphocyte composition. These transformed variables, rather than the unprocessed raw percentages, were used in the subsequent variable−selection and regression−modeling procedures. Routine hematological and clinicopathological variables were retained in their original scale or transformed when clinically or statistically appropriate.

### 2.4. Data Partitioning

The original dataset was divided into training and independent validation sets at a 7:3 ratio using stratified random sampling. Stratification was performed according to peritoneal metastasis status to ensure a similar proportion of PM cases in both subsets. Finally, 303 cases were assigned to the training set and 130 cases to the independent validation set. The validation set was kept separate during model development and was used only for model evaluation.

### 2.5. Preprocessing of Lymphocyte Subset Variables and LASSO Regression Analysis

LASSO regression was used for variable selection and the handling of high−dimensional data. This method applies L1 regularization to the regression coefficients, shrinking some coefficients to zero, thereby simplifying the model and variable set. In this study, all variables were entered into the LASSO regression model with peritoneal metastasis as the dependent variable. The optimal regularization parameter (λ) was determined using a 10−fold cross−validation. We selected the λ value corresponding to the minimum mean squared error (MSE; λ.min) and the λ value within one standard error of the minimum error for the simplified model (λ.1se). Based on the λ.1se threshold, 7 variables with nonzero coefficients were retained in the model. Variance inflation factor (VIF) analysis was performed to evaluate multicollinearity among candidate predictors. Variables with VIF >5 were considered to have substantial collinearity.

### 2.6. Logistic Regression Analysis

The LASSO−selected candidate predictors were further evaluated using logistic regression in the training cohort. Univariable logistic regression was performed as a descriptive analysis for each LASSO−selected predictor. All seven LASSO−selected candidate predictors were then entered into the multivariable logistic regression model. The final model retained predictors that remained independently associated with peritoneal metastasis in the multivariable analysis. Odds ratios and 95% confidence intervals were reported for each predictor.

### 2.7. Model Performance Evaluation and Internal Validation

The final model was developed in the training cohort and evaluated in the randomly held−out internal validation cohort. The validation cohort was not used for variable selection, model fitting, or model optimization. Discrimination was assessed using the area under the receiver operating characteristic curve (AUROC) and the area under the precision−recall curve (AUPRC). The 95% confidence intervals for AUROC and AUPRC were estimated using 2000 patient−level bootstrap resamples of the validation cohort without refitting the model. The optimal probability threshold was determined in the training cohort by maximizing the Youden index, defined as *J* = sensitivity + specificity − 1. This threshold was then applied unchanged to the validation cohort. Sensitivity, specificity, accuracy, positive predictive value, and negative predictive value were calculated in the validation cohort at both the conventional threshold of 0.50 and the Youden−derived threshold. Calibration was evaluated in the validation cohort using calibration curves, the Brier score, calibration intercept, and calibration slope. The Brier score was calculated as the mean squared difference between the predicted probability and the observed outcome. Clinical utility was assessed using decision curve analysis in the validation cohort.

### 2.8. Visualization and Interpretability

A nomogram was constructed based on the final multivariable logistic regression model to provide individualized estimation of the probability of peritoneal metastasis. Model interpretability was further evaluated using SHAP analysis. Because the final prediction model was a logistic regression model, linear SHAP values were calculated to quantify the contribution of each predictor to the predicted risk for individual patients.

A nomogram was constructed from the final multivariable logistic regression model fitted in the training cohort. SHAP values were calculated using the held−out validation cohort to interpret the predictions of the final model in patients who were not used for model development. SHAP analysis was performed using the R package “shapviz” [13].

### 2.9. Subgroup Analysis

In the multivariable logistic regression analysis, NK cells and NKT−like cells were identified as independent predictors of peritoneal metastasis. Because the subgroup analysis was intended to describe the observed distribution of NK and NKT−like cell percentages in the original patient population, raw percentages were used for clinical interpretability. In contrast, log−ratio−transformed immune−cell variables were used in LASSO and multivariable logistic regression to address the compositional nature of flow cytometry−derived lymphocyte subset data. Therefore, subgroup analysis was conducted for these two cell populations to investigate their differential expression profiles. Between−group differences were compared using the Mann–Whitney U test. Spearman’s rank correlation test was used to assess the relationship between NK and NKT−like cells. The results are presented as violin plots, ROC curves, and scatter plots with fitted regression lines. Furthermore, the diagnostic performance of NK cells, NKT−like cells, and the NK/NKT−like cells ratio was evaluated using ROC curve analysis.

### 2.10. Statistical Analysis

Statistical significance was defined as a two−sided *p* value < 0.05. All statistical analyses were performed using SPSS version 27.0 (IBM Corp., Armonk, NY, USA), R version 4.5 (R Foundation for Statistical Computing, Vienna, Austria), and GraphPad Prism version 10 (GraphPad Software, San Diego, CA, USA). Continuous variables that conformed to a normal distribution were presented as the mean ± standard deviation (SD), and between−group differences were analyzed using an independent−samples *t*−test. Continuous variables with non−normal distributions were presented as the median and interquartile range (IQR) and compared using the Mann–Whitney U test. Categorical variables were presented as counts and percentages (%) and compared using the chi−squared test or Fisher’s exact test.

## 3. Results

### 3.1. Patient Characteristics

Among the 433 included patients, 93 were found to have macroscopic PM during surgical exploration and underwent exploratory laparotomy without curative gastrectomy, whereas 340 patients had no evidence of PM and proceeded to gastrectomy according to the planned surgical strategy. In this study, including 303 men (69.98%) and 130 women (30.02%). Among them, 137 patients (31.64%) were aged ≥65 years, and 296 patients (68.36%) were aged <65 years; 405 patients (93.53%) had an ECOG performance status of 0–1. Significant differences were observed in multiple hematological parameters (*p* < 0.05); however, no statistically significant differences were found in sex, age, body mass index (BMI), γ−GGT, or other biochemical parameters (Table 1).

### 3.2. LASSO Regression Analysis

**Figure 2:**
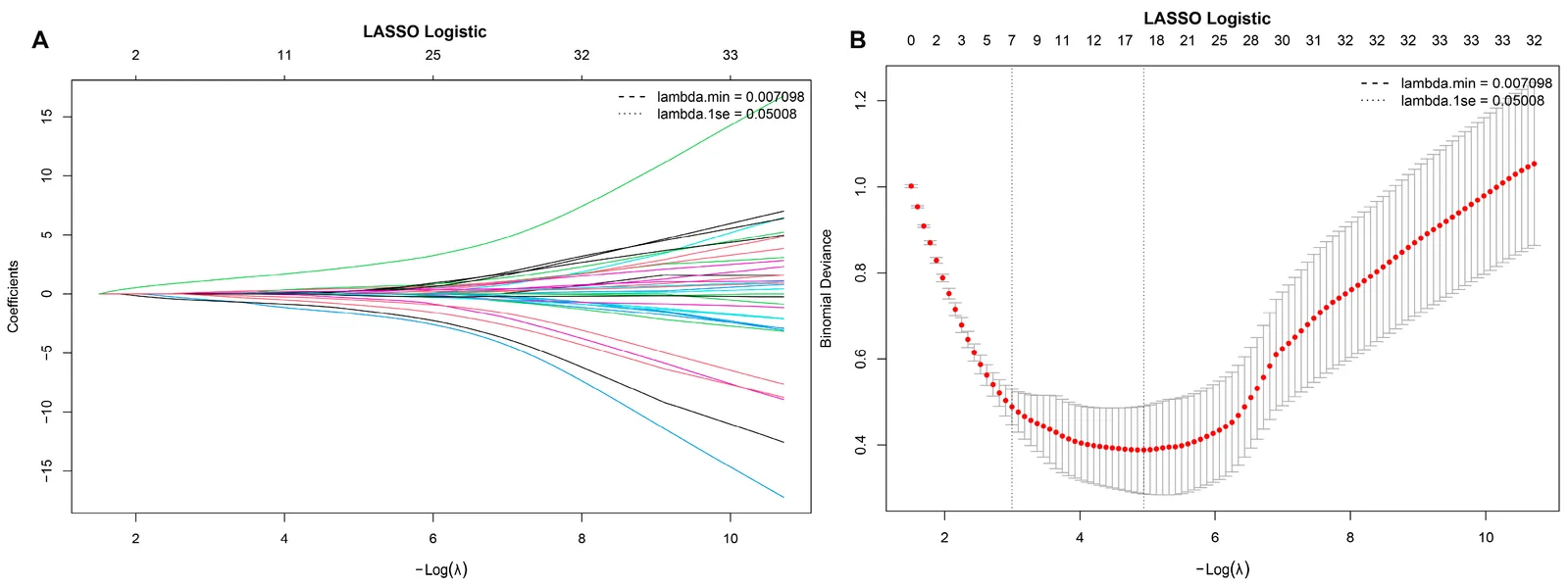
LASSO regression for variable selection. (**A**) Coefficient shrinkage of all candidate paths of all candidate variables as a function of −log(λ). (**B**) 10−fold cross−validation curve for λ selection.

To identify the predictors of peritoneal metastasis, variables were analyzed using the LASSO regression model. All candidate variables were screened using LASSO regression with 10−fold cross−validation (Figure 2), and the regularization parameter λ was selected based on λ.1se. Finally, DBIL, PALB, CA125, LDH, LYM, NK cell and NKT−like cell variables with non−zero β−coefficients were identified.

### 3.3. Logistic Regression

Perform univariable logistic regression on 7 variables. Univariable logistic regression analysis identified 7 univariable significantly associated with peritoneal metastasis (all *p* < 0.05). These variables included log(NKT−like cells/remaining lymphocytes) (OR = 12.105, 95%CI: 8.322–15.156, *p* < 0.001), DBIL (OR = 0.421, 95% CI: 0.314–0.547, *p* < 0.001), PALB (OR = 0.981, 95% CI: 0.976–0.986, *p* < 0.001), log(NK cells/remaining lymphocytes) (OR = 0.062, 95% CI: 0.017–0.201, *p* < 0.001), LYM (OR = 0.255, 95% CI: 0.145–0.428, *p* < 0.001), LDH (OR = 1.016, 95% CI: 1.010–1.021, *p* < 0.001), and CA125 (OR = 1.020, 95% CI: 1.010–1.033, *p* < 0.001) (Table 2).

After the 7 variables were incorporated into the multivariable logistic regression model, 7 variables remained independently associated with PM, among which log(NKT−like cells/remaining lymphocytes) (OR = 15.004, 95%CI: 11.732–24.630, *p* < 0.001) showed the strongest predictive association (OR = 15.004) (Table 2). DBIL, PALB, log(NK cells/remaining lymphocytes) and LYM may be inversely associated protective factors.

### 3.4. Model Performance Evaluation

**Figure 3:**
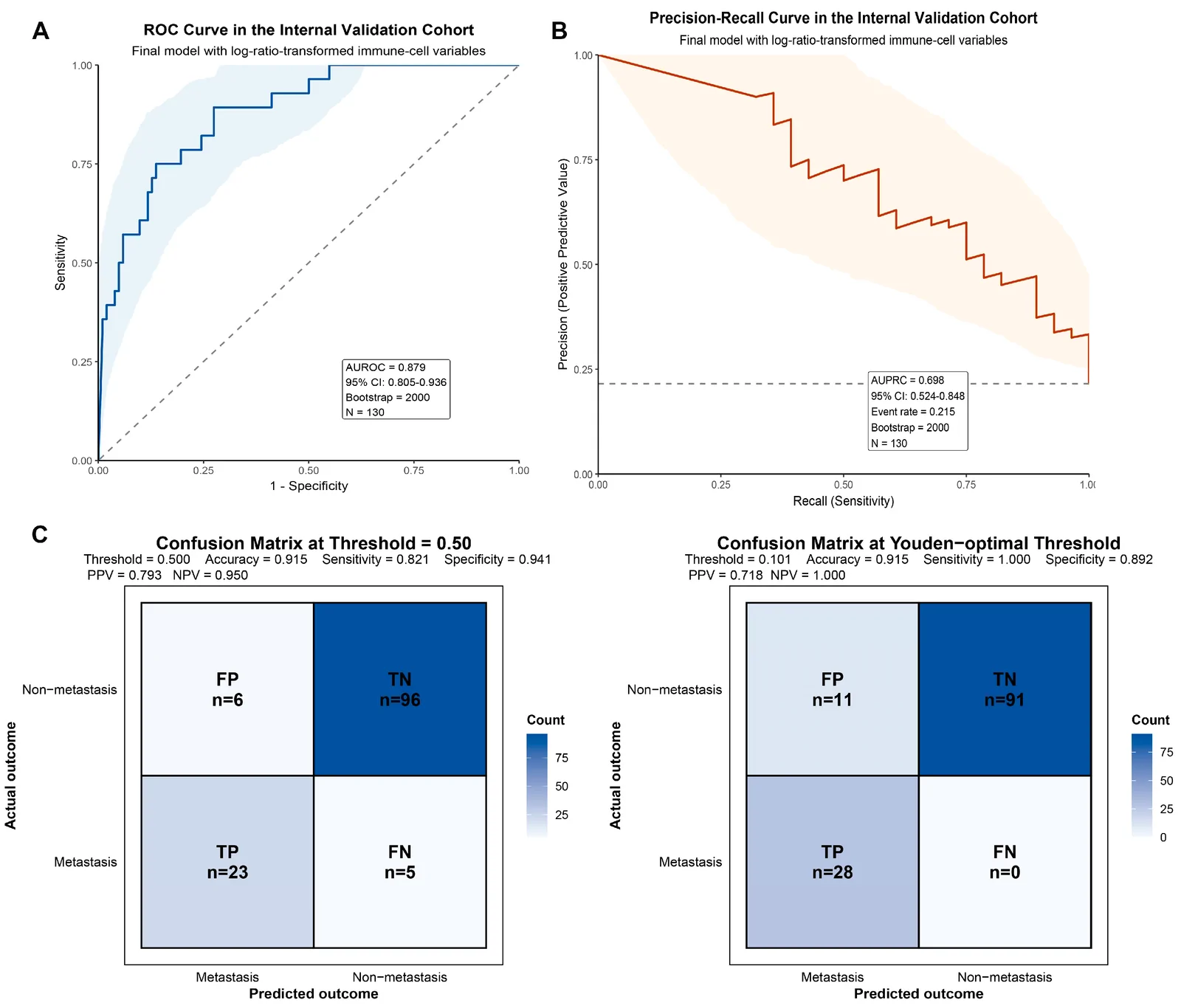
Performance of the final multivariable logistic regression model with log–ratio–transformed immune−cell variables in the held−out internal validation cohort (*n* = 130). (**A**) Receiver operating characteristic curve and AUROC. (**B**) Precision–recall curve and AUPRC. (**C**) Confusion matrices at the conventional probability threshold of 0.50 and the Youden–optimal threshold of 0.101. Rows represent the observed outcome, columns represent the predicted class, and PM was defined as the positive class.

The final multivariable logistic regression model incorporating log−ratio−transformed immune−cell variables was evaluated in the randomly held−out internal validation cohort, which included 130 patients, comprising 28 patients with PM and 102 patients without PM. The model demonstrated good discriminative ability, with an AUROC of 0.879 (95% CI: 0.805−0.936; Figure 3A). Because PM represented the minority outcome, precision–recall analysis was additionally performed. The model achieved an AUPRC of 0.698 (95% CI: 0.524−0.848), which was substantially higher than the baseline PM prevalence of 21.5% in the validation cohort, indicating satisfactory discrimination for the minority class (Figure 3B).

At the conventional probability threshold of 0.500, the model achieved an accuracy of 0.915, sensitivity of 0.821, specificity of 0.941, positive predictive value (PPV) of 0.793, and negative predictive value (NPV) of 0.950. Using the Youden−optimal cutoff value, the sensitivity, specificity, accuracy, PPV, and NPV were 1.000, 0.892, 0.915, 0.718, and 1.000, respectively. The corresponding confusion matrices are presented in Figure 3C. Additional calibration and decision curve analyses are provided in Supplementary Figure S1.

### 3.5. Model Visualization

**Figure 4:**
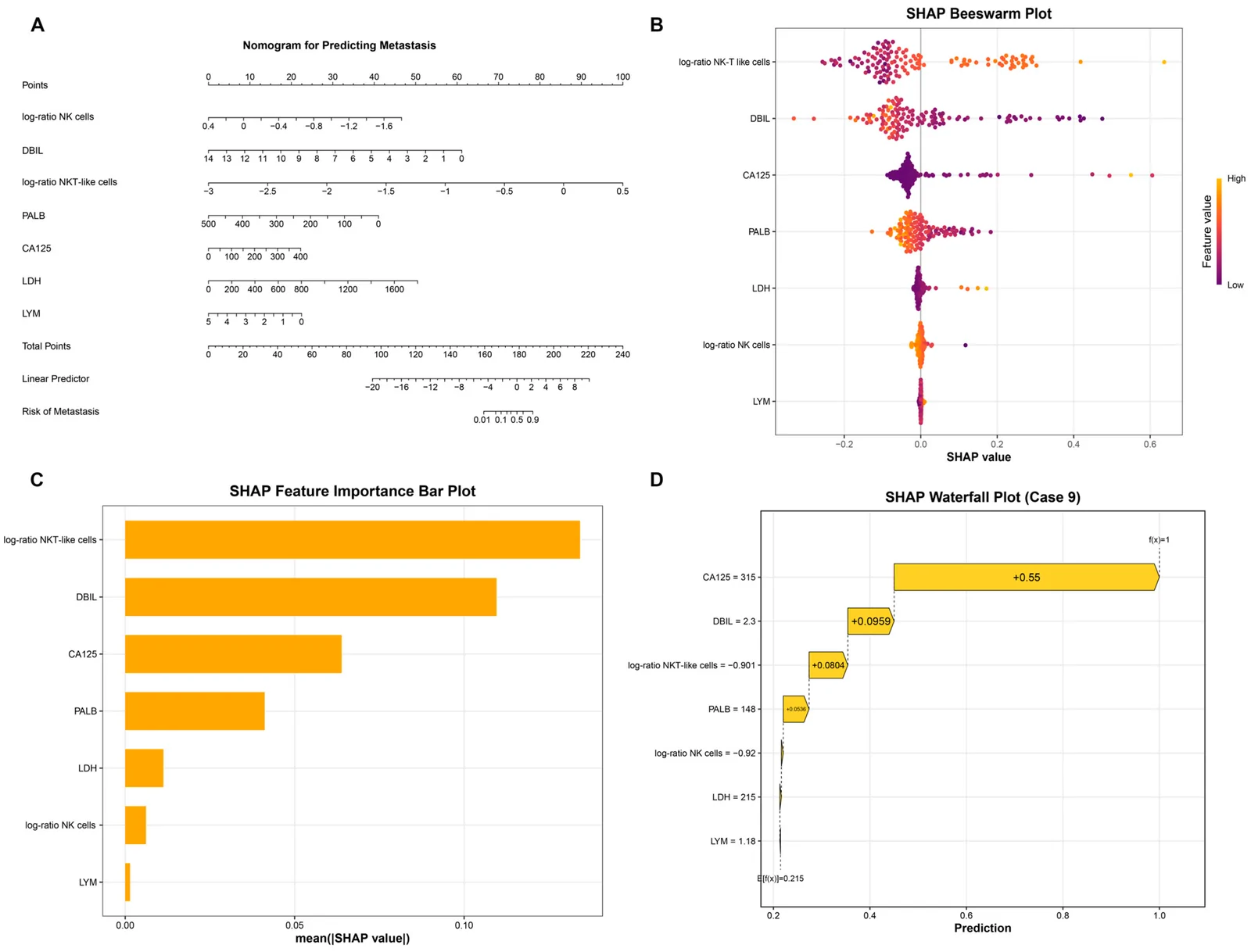
Nomogram and SHAP interpretability analysis of the final prediction model based on 7 independent predictors. (**A**) Nomogram for the individualized prediction of peritoneal metastasis probability (*n* = 303); (**B**) SHAP beeswarm plot; (**C**) SHAP feature importance bar plot; and (**D**) SHAP waterfall plot for a representative high–risk case.

A nomogram was developed using 7 independent predictors to support the individualized assessment of peritoneal metastasis risk (Figure 4A). SHAP analysis identified NKT−like cells, NK cells, DBIL, and PALB as the main contributors to the model prediction (Figure 4B,C). The swarm plot showed that DBIL, PALB, and NKT−like cells were negatively associated with PM prediction, whereas NK cells contributed positively.

### 3.6. Exploratory Analysis of NK and NKT−like Cell Profiles

**Figure 5:**
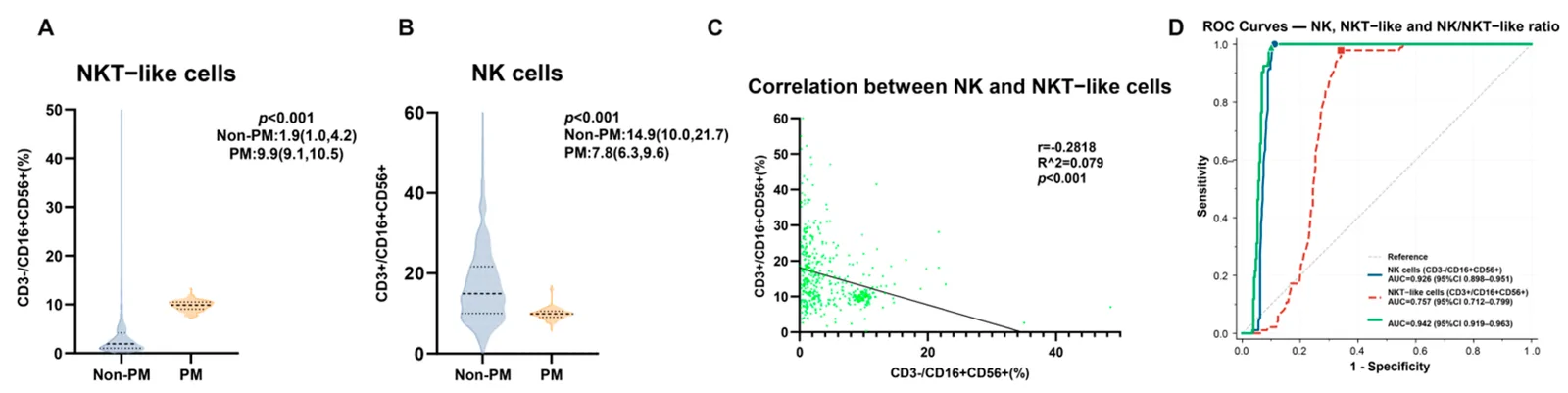
Exploratory subgroup analysis of raw NK and NKT−like cell percentages in the original full cohort without SMOTE augmentation (*n* = 433). (**A**) Distribution of NKT−like cell percentages in the PM and non−PM groups; (**B**) Distribution of NK cell percentages in the PM and non−PM groups; (**C**) Spearman correlation between NK and NKT−like cell levels; and (**D**) Receiver operating characteristic (ROC) curves for the diagnosis of peritoneal metastasis using NK cells, NKT−like cells, and the NK/NKT−like ratio.

NK cells and NKT−like cells showed opposite predictive patterns for PM. Therefore, subgroup analyses were performed to further examine their expression patterns and diagnostic value. NKT–like cell levels were significantly higher in patients with PM than in those without PM (9.9 [9.1–10.5] vs. 1.9 [1.0–4.2], *p* < 0.001, Figure 5A), whereas NK cell levels were significantly higher in patients without PM than in those with PM (14.9 [10.0–21.7] vs. 7.8 [6.3–9.6], *p* < 0.001, Figure 5B). Correlation analysis showed a significant negative correlation between NK cells and NKT−like cells (r = −0.314, *p* < 0.001, Figure 5C). ROC curve analysis further showed that NK cells (AUC = 0.926, 95% CI: 0.898–0.951), NKT−like cells (AUC = 0.757, 95% CI: 0.712–0.799), and the NK/NKT ratio (AUC = 0.942, 95% CI: 0.919–0.963) had diagnostic value for PM, with the NK/NKT ratio showing the strongest discriminative ability (Figure 5D). To further address the compositional nature of flow cytometry−derived lymphocyte subset variables, corresponding subgroup analyses were performed in the log−ratio space using the original full cohort. The log–ratio NK–cell variable was lower in patients with PM than in those without PM (median, −0.8479 vs. −0.6711; *p* < 0.001; Supplementary Figure S2A). In contrast, the log–ratio NKT–like–cell variable was higher in patients with PM than in those without PM (median, −0.8529 vs. −1.556; *p* < 0.001; Supplementary Figure S2B). The correlation between the two transformed variables was weak (r = −0.1627, *p* = 0.005; Supplementary Figure S2C). ROC analysis showed that the log−ratio NKT–like–cell variable had good discriminative ability for PM (AUC = 0.926, 95% CI: 0.895–0.957), whereas the log−ratio NK−cell variable showed moderate discrimination (AUC = 0.727, 95% CI: 0.672–0.781). A combined log−ratio immune−cell score showed the highest discrimination (AUC = 0.946, 95% CI: 0.919–0.973; Supplementary Figure S2D).

## 4. Discussion

In this study, LASSO regression was first applied to screen the variables with nonzero β coefficients. These variables were combined with key clinical features to construct a logistic regression model that included 7 candidate predictors. The model exhibited excellent predictive performance with an AUC of 0.879. Notably, the NK/NKT−like cell profile was a useful predictor of peritoneal metastasis in patients with GC. To further support clinical interpretation, we developed a nomogram and SHAP plots to visualize the model results.

Multivariable logistic regression analysis showed that DBIL and PALB were independently associated with peritoneal metastasis in GC. Previous studies have also reported a significant association between bilirubin and GC [14]. Bilirubin may help suppress tumor formation by reducing oxidative stress, regulating proto−oncogene/oncogene signaling pathways, and limiting immune evasion [15]. PALB is mainly synthesized in the liver and is commonly regarded as a sensitive marker of nutritional and inflammatory status [16]. In addition, a multicenter retrospective study found that higher albumin levels independently predicted better overall survival and progression−free survival [17]. In our study, PALB levels were significantly lower in the PM group than in the non−PM group, consistent with previous reports [18,19,20]. These findings suggest that liver−derived inflammatory and nutritional biomarkers may help predict PM status and support preoperative risk assessment in patients with GC.

In the subgroup analyses, NK and NKT−like cells showed a negative correlation. Tumor cells may escape NK cell−mediated cytotoxicity by binding to inhibitory receptors on NK cells. In addition, excessive production of TGF−β [21] and other anti−inflammatory cytokines and chemokines in the tumor microenvironment can suppress NK cell activation and downregulate NK cell−activating receptors and co−receptors [22]. GC cells may also impair NK cell survival through l−kynurenine, a product of indoleamine 2,3−dioxygenase activity [8]. Together, these findings suggest that NK cells may increase in number as a compensatory response but remain functionally exhausted. Peng et al. reported that CD3+CD56+ NKT−like cells were significantly reduced in GC tumors, and lower infiltration of these cells was associated with poorer survival and disease progression [23]. In contrast, type I NKT−like cells showed strong antitumor activity against CD1d−positive GC cells in the presence of α−GalCer, both in vitro and in vivo [24]. Consistent with these reports, our study showed that peripheral NK cells were significantly lower in the PM group than in the non−PM group. These findings may reflect alterations in immune cell composition during peritoneal dissemination; however, because lymphocyte subsets represent compositional data, the observed correlation should be interpreted cautiously.

The “black box” nature of complex algorithms remains a major barrier to the clinical use of predictive models in oncology [13]. To improve interpretability, we used SHAP analysis to break down each prediction into the additive contribution of individual features within a cooperative game−theoretic framework [25]. In our cohort, DBIL, PALB, LYM, LDH, CA125, NK cells, and NKT−like cells showed substantial SHAP values, which were consistent with their multivariable odds ratios and supported the robustness of the model. The waterfall plot further showed the direction and magnitude of each feature’s contribution to individual predictions. These case−level explanations help clarify how the model makes decisions and provide traceable evidence for individual risk assessment, which is particularly important in clinical settings where transparency strongly influences trust in algorithms [26].

Previous retrospective studies on peritoneal metastasis in GC have rarely included preoperative flow cytometry data. In our model, flow cytometry−−derived NK/NKT cell profiles showed good predictive value. This is different from most existing models, which mainly depend on tumor markers and imaging findings. By incorporating flow cytometry data, our study provides additional information on the tumor immune microenvironment and may help further explore immune−related mechanisms.

Several limitations should be noted. First, this was a single−center retrospective study; therefore, selection bias cannot be avoided. Further multicenter prospective studies are needed to confirm the generalizability of our findings. Second, no external validation cohort was included, and the model was validated only by internal bootstrap resampling. Third, the relatively small sample size may have limited the statistical power of the subgroup analyses. Fourth, this retrospective study could not establish causal relationships. The functional status of NK/NKT−like cells may provide additional predictive information and should be evaluated in future prospective functional studies. Finally, several clinically important factors, including laparoscopic staging, Lauren classification, and vascular invasion, were not included in the analysis.

## 5. Conclusions

The multivariable logistic regression model developed in this study, based on LASSO−selected variables, showed promising discrimination in an internal hold−out cohort. Our findings suggest that NK and NKT−like cells have important predictive value and provide a new immune microenvironment−based perspective for PM prediction. In addition, the nomogram and SHAP visualizations generated from the model may help clinicians perform individualized risk stratification for PM.

## Figures and Tables

**Table 1:** Patients’ characteristics.

Characteristics	Training Set		Independent Validation Set		*p*-Value
	PM	Non−PM	PM	Non−PM	
	(*n* = 65)	(*n* = 238)	(*n* = 28)	(*n* = 102)	
Gender					0.591
Female	16 (24.615%)	70 (29.412%)	9 (32.143%)	35 (34.137%)	
Male	49 (75.385%)	168 (70.588%)	19 (67.857%)	67 (65.686%)	
Age					0.147
<65	37 (56.923%)	164 (68.908%)	21 (75%)	74 (72.549%)	
≥65	28 (43.077%)	74 (31.092%)	7 (25%)	28 (27.451%)	
BMI (Mean ± SD)	23.09 (3.643)	22.73 (3.136)	22.32 (3.1)	22.62 (3.589)	0.737
ECOG					<0.001
0–1	46 (70.769%)	236 (99.160%)	23 (82.143%)	100 (98.039%)	
≥2	19 (29.231%)	2 (0.840%)	5 (17.857%)	2 (1.961%)	
ALT (IQR)	12 (9, 16.75)	18 (13.75, 24)	10.5 (8, 14.75)	20 (14, 25.25)	<0.001
AST (IQR)	15.5 (14, 19)	20 (16.75, 25)	16.5 (14, 19.75)	20 (16, 26.25)	<0.001
γ−GGT (IQR)	17 (13, 27.75)	17 (11, 26)	15 (13, 23.75)	16.5 (12, 27.25)	0.717
LDH (IQR)	238.5 (188.5, 277.3)	159 (143, 178)	203.5 (187, 218.8)	160 (143, 185.5)	<0.001
TBIL (IQR)	10.6 (7.775, 14.57)	10.73 (8.455, 15.12)	11.05 (8.325, 15.33)	11.68 (8.945, 16.17)	0.690
DBIL (IQR)	2.2 (1.5, 2.875)	3.93 (2.86, 5)	2.35 (1.6, 3.675)	4.155 (3.23, 5.3)	<0.001
IDBIL (IQR)	8.4 (6.35, 11.95)	7.14 (5.225, 10.19)	8.65 (6.725, 8.65)	7.105 (5.313, 10.49)	0.002
TP (IQR)	62.25 (56.7, 66.78)	67 (63, 71)	62.45 (58.53, 67.95)	68 (64.75, 72.13)	<0.001
ALB (IQR)	35.95 (32.75, 39.5)	41 (38, 43)	37.95 (34.9, 41)	41 (38.75, 44)	<0.001
GLOB (IQR)	25.65 (22.5, 28.5)	26 (24, 29)	24.45 (22.43, 26.48)	27 (24, 30)	0.020
A/G (IQR)	1.4 (1.2, 1.6)	1.5 (1.4, 1.7)	1.6 (1.4, 1.8)	1.5 (1.3, 1.7)	0.020
PALB (IQR)	187 (142.8, 232.3)	272.5 (227.5, 315)	194 (149.5, 241)	264 (217.8, 316.3)	<0.001
UREA (IQR)	5.25 (4, 6.55)	5.6 (4.5, 6.9)	5.15 (3.925, 5.975)	5.8 (5, 6.925)	0.04
CREA (IQR)	77 (64.25, 87.5)	82 (74, 92)	72.1 (67.05, 82.93)	82.5 (72, 92)	<0.001
UA (IQR)	270.5 (202.5, 325)	299 (243.8, 353.3)	262.5 (220.8, 320.5)	305 (250.8, 346.3)	0.02
ALP (IQR)	80.5 (62.25, 95)	73 (61.75, 87.25)	75 (57.5.96.75)	71 (59.75, 91)	0.672
Glu (IQR)	5.2 (4.7, 5.875)	5 (4.6, 5.5)	4.95 (4.225, 5.6)	5 (4.575, 5.7)	0.256
WBC (IQR)	6.58 (5.758, 8.575)	6.315 (5.19, 7.703)	5.65 (4.483, 6.795)	6.68 (5.328, 7.398)	0.023
NEU (IQR)	4.6 (3.51, 6.1)	3.63 (2.74, 4.605)	3.32 (2.315, 4.758)	3.59 (2.978, 4.523)	<0.001
LYM (Mean ± SD)	1.471 (0.5642)	1.974 (0.6783)	4.523 (0.6644)	2 (0.822)	<0.001
MONO (IQR)	0.5 (0.4, 0.65)	0.47 (0.3675, 0.615)	0.385 (0.3225, 0.5025)	0.455 (0.35, 0.61)	0.040
EOSI (IQR)	0.1 (0.0625, 0.2075)	0.125 (0.0675, 0.22)	0.09 (0.04, 0.1375)	0.135 (0.088, 0.2025)	0.047
Hb (IQR)	126 (101, 139.8)	137 (123, 155)	129.5 (97, 138.8)	138.8 (126.7, 151.3)	<0.001
RBC (Mean ± SD)	4.174 (0.6228)	4.418 (0.63)	4.055 (0.7385)	4.51 (0.4876)	<0.001
HCT (IQR)	37.95 (31.9, 41.25)	40.84 (37.21, 45.53)	38.4 (29.63, 40.58)	41.6 (38.2, 44.43)	<0.001
Plt (IQR)	268 (218.5, 329.3)	248 (198.8, 296.3)	256.5 (226.3, 330)	238 (204.8, 284.5)	0.093
CEA (IQR)	2.485 (1.31, 10.12)	1.88 (1.215, 3.003)	1.91 (0.9, 7.398)	2.1 (1.398, 4.53)	0.027
CA199 (IQR)	17.39 (6.913, 59.29)	10.19 (6.165, 16.62)	24 (4.4, 74.73)	9.92 (6.018, 19.34)	0.001
CA724 (IQR)	7.215 (3.303, 12.64)	2.182 (1.17, 5.898)	7.015 (2.29, 17.23)	1.985 (1.090, 4.098)	<0.001
CA125 II (IQR)	22.78 (9.938, 53.45)	10.22 (7.393, 14.16)	23.32 (10.88, 42.37)	10.18 (7.395, 14.58)	<0.001
CD3+ (IQR)	63.2 (53.18, 71.6)	69.8 (62.2, 74.95)	62.7 (55.75, 73.8)	69.4 (64.08, 75.7)	0.040
CD3+/CD4+ (IQR)	42.4 (33.73, 47.78)	41 (34.68, 45.9)	40.25 (34, 47.43)	40.95 (35.08, 46.8)	0.923
CD3+/CD8+ (IQR)	23.3 (16.65, 35.3)	22.7 (17.55, 28.43)	22.05 (15.8, 36.88)	22.35 (17.1, 29.15)	0.766
CD4+/CD8+ (IQR)	1.695 (1.13, 2.268)	1.87 (1.34, 2.505)	1.665 (1.215, 2.013)	1.86 (1.278, 2.513)	0.049
CD3+/CD4+CD8+ (IQR)	0.3 (0.2, 0.5)	0.3 (0.1, 0.5225)	0.4 (0.225, 0.575)	0.3 (0.1, 0.6)	0.178
CD19+ (IQR)	12 (7.225, 15.38)	11.05 (8, 14.33)	12.6 (7.05, 16.85)	10.7 (8.15, 13.55)	0.647
CD3−/CD16+CD56+ (IQR)	9.85 (8.9, 10.58)	14.85 (10, 21.45)	9.85 (9.25, 10.5)	15.15 (9.65, 22.03)	<0.001
CD3+/CD16+CD56+ (IQR)	9.9 (9.125, 10.58)	2 (1, 3.725)	9.8 (8.6, 10.38)	1.9 (1.1, 4.625)	<0.001

**Table 2:** Univariable and multivariable logistic regression analysis (*n* = 7).

Characteristics	Univariable Analysis		Multivariable Analysis	
	OR (95% CI)	*p*-Value	OR (95% CI)	*p*-Value
log(NKT−like cells/remaining lymphocytes)	12.105(8.322–15.156)	<0.001	15.004(11.732–24.630)	<0.001
DBIL	0.421(0.314–0.547)	<0.001	0.352(0.200–0.552)	<0.001
PALB	0.981(0.976–0.986)	<0.001	0.981(00968–0.991)	<0.001
log(NK cells/remaining lymphocytes)	0.062(0.017–0.201)	<0.001	0.006(0.001–0.106)	0.002
LYM	0.255(0.145–0.428)	<0.001	0.341(0.117–0.904)	0.036
LDH	1.016(1.010–1.021)	<0.001	1.006(1.002–1.010)	<0.001
CA125	1.020(1.010–1.033)	<0.001	1.013(1.002–1.026)	0.044

## Data Availability

The datasets used and analyzed during the current study are available from the corresponding author on reasonable request.

## Supplementary Materials

The following supporting information can be downloaded at: https://www.mdpi.com/article/10.3390/cancers18172813/s1, Figure S1: Additional performance assessment of the final model in the held−out internal validation cohort. (A) Calibration curve. (B) Decision curve analysis. Figure S2: Exploratory subgroup analysis of log−ratio−transformed NK and NKT−like cell variables in the original full cohort (*n* = 433). (A) Distribution of the log−ratio NK−cell variable between patients with and without peritoneal metastasis. (B) Distribution of the log−ratio NKT−like−cell variable between patients with and without peritoneal metastasis. (C) Correlation between log−ratio NK−cell and NKT−like−cell variables. (D) ROC curves for the log−ratio NK−cell variable, log−ratio NKT−like−cell variable, and a combined log−ratio immune−cell score. These analyses were performed for supplementary visualization and were not used for final model fitting or validation.

## Abbreviations

ALB, albumin; ALP, alkaline phosphatase; ALT, alanine aminotransferase; AST, aspartate aminotransferase; AUROC, area under the receiver operating characteristic curve; BMI, body mass index; CA125, carbohydrate antigen 125; CA199, carbohydrate antigen 19−9; CA724, carbohydrate antigen 72−4; CEA, carcinoembryonic antigen; CI, confidence interval; CREA, creatinine; DBIL, direct bilirubin; DCA, decision curve analysis; ECOG, Eastern Cooperative Oncology Group performance status; GC, gastric cancer; GGT, gamma−glutamyl transferase; GLU, glucose; Hb, hemoglobin; IDBIL, indirect bilirubin; IQR, interquartile range; LASSO, least absolute shrinkage and selection operator; LDH, lactate dehydrogenase; LYM, lymphocyte count; Neu, neutrophil count; NK, natural killer; NKT, natural killer T; NPV, negative predictive value; OR, odds ratio; PALB, prealbumin; PLT, platelet count; PM, peritoneal metastasis; PPV, positive predictive value; RBC, red blood cell count; RCS, restricted cubic spline; SD, standard deviation; SHAP, SHapley Additive exPlanations; SMOTE, Synthetic Minority Over−sampling Technique; TP, total protein; UA, uric acid; UREA, blood urea nitrogen; WBC, white blood cell count.
