# Supplementary material for: A Logistic Regression Model Integrating Flow Cytometry−Derived Immune Cell Profiles and Hematological Parameters for Preoperative Prediction of Peritoneal Metastasis in Gastric Cancer
Source: Cancers (Basel). 2026 Aug 30;18(17):2813. doi: 10.3390/cancers18172813 (PMC13565575; doi:10.3390/cancers18172813)
Supplement: Supplementary file 1 [file cancers-18-02813-s001.zip › cancers-4383471-supplementary.pdf]

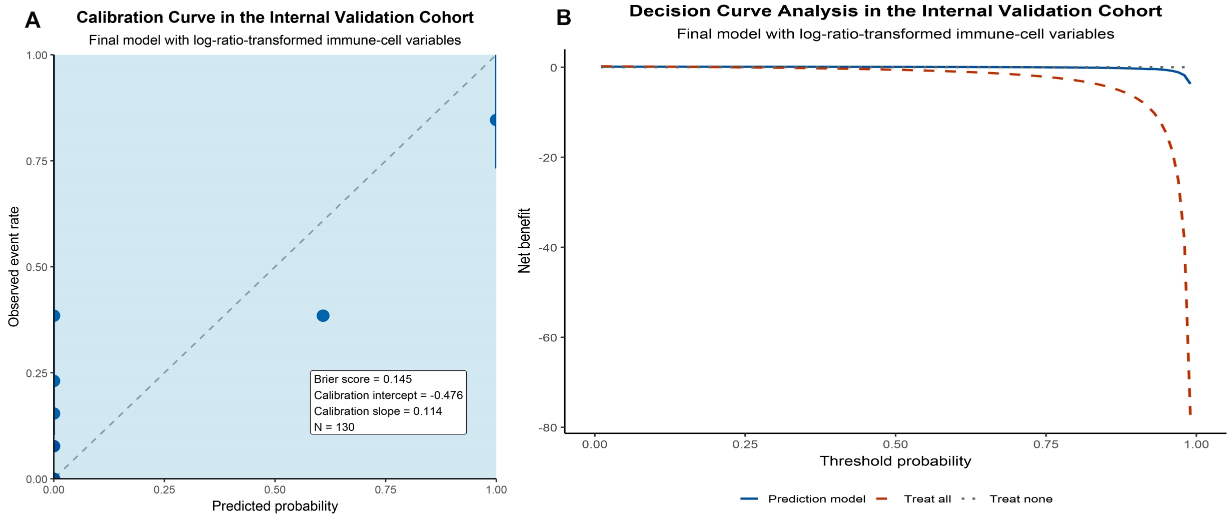

Supplementary Figure S1. Additional performance assessment of the final model in the held-out internal validation cohort. (A) Calibration curve. (B) Decision curve analysis.

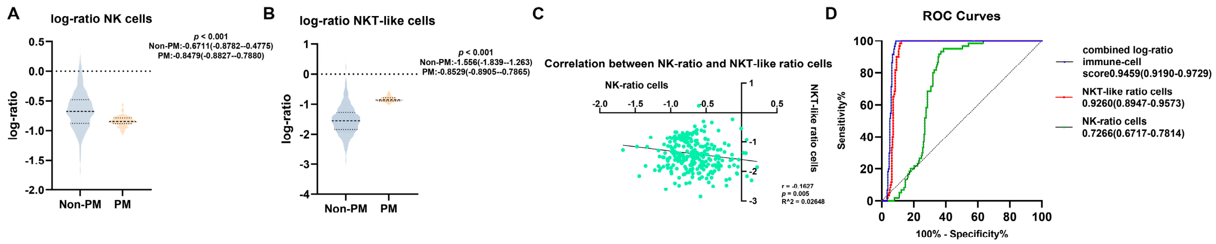

Supplementary Figure S2. Exploratory subgroup analysis of log-ratio-transformed NK and NKT-like cell variables in the original full cohort (n = 433). (A) Distribution of the log-ratio NK-cell variable between patients with and without peritoneal metastasis. (B) Distribution of the log-ratio NKT-like-cell variable between patients with and without peritoneal metastasis. (C) Correlation between log-ratio NK-cell and NKT-like-cell variables. (D) ROC curves for the log-ratio NK-cell variable, log-ratio NKT-like-cell variable, and a combined log-ratio immune-cell score. These analyses were performed for supplementary visualization and were not used for final model fitting or validation.
